# Supplementary material for: PIEZO1-mediated mechanosensation links aging to bladder dysfunction
Source: bioRxiv. 2026 May 25:2026.05.24.726025. Preprint. [Version 1] doi: 10.64898/2026.05.24.726025 (PMC13232120; doi:10.64898/2026.05.24.726025)
Supplement: Supplement 1 [file NIHPP2026.05.24.726025v1-supplement-1.pdf]

bioRxiv preprint doi: https://doi.org/10.64898/2026.05.24.726025; this version posted May 25, 2026. The copyright holder for this preprint (which was not certified by peer review) is the author/funder, who has granted bioRxiv a license to display the preprint in perpetuity. It is made available under a CC-BY 4.0 International license.

SUPPLEMENTARY FIGURES

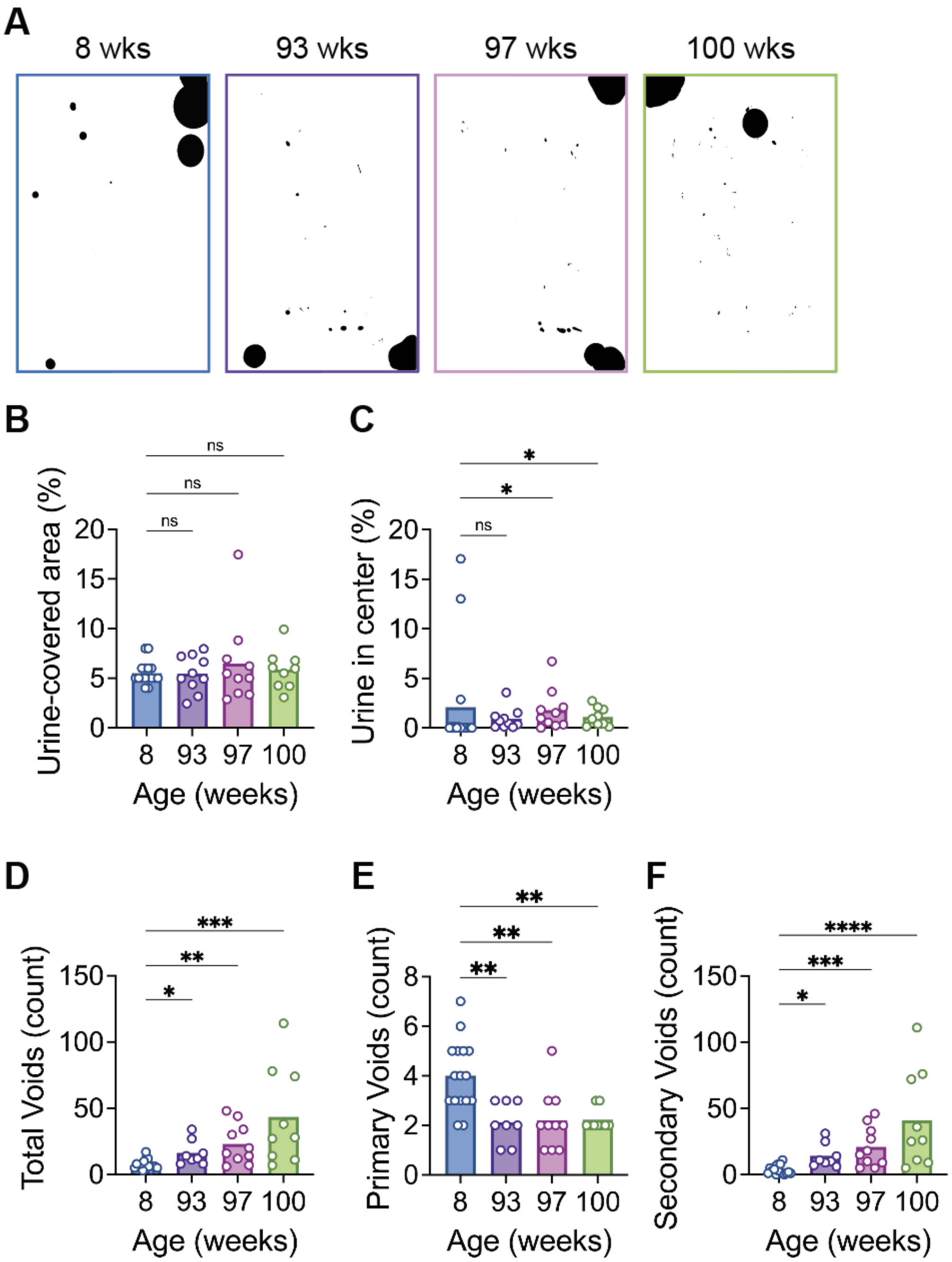

Figure S1. Aging-related changes in urinary behavior are more subtle in aged female mice than male mice.

(A) Representative images of 4h VSA papers from young (8-week-old) and aged (93-, 97-, and 100-week-old) female mice. Urine void spots are shown in black.

(B) Total urine-covered area at the end of a 4 h void spot assay, quantified as percentage of total cage area; n=9-16/group.

(C) Percentage of urine deposited in center 25% of cage area; n=9-16/group.

(D) Total number of urinary void spots, further stratified by size into primary (E) and secondary (F); n=9-16/group.

For all bar plots: bars show mean; dots show individual animals. Statistical test: (B-F) Kruskal-Wallis test with Dunn's multiple comparisons ad hoc test. (ns) $p > 0.05$, (*) $p \leq 0.05$, (**) $p \leq 0.01$, (***) $p \leq 0.001$, (****) $p \leq 0.0001$.

bioRxiv preprint doi: https://doi.org/10.64898/2026.05.24.726025; this version posted May 25, 2026. The copyright holder for this preprint (which was not certified by peer review) is the author/funder, who has granted bioRxiv a license to display the preprint in perpetuity. It is made available under a CC-BY 4.0 International license.

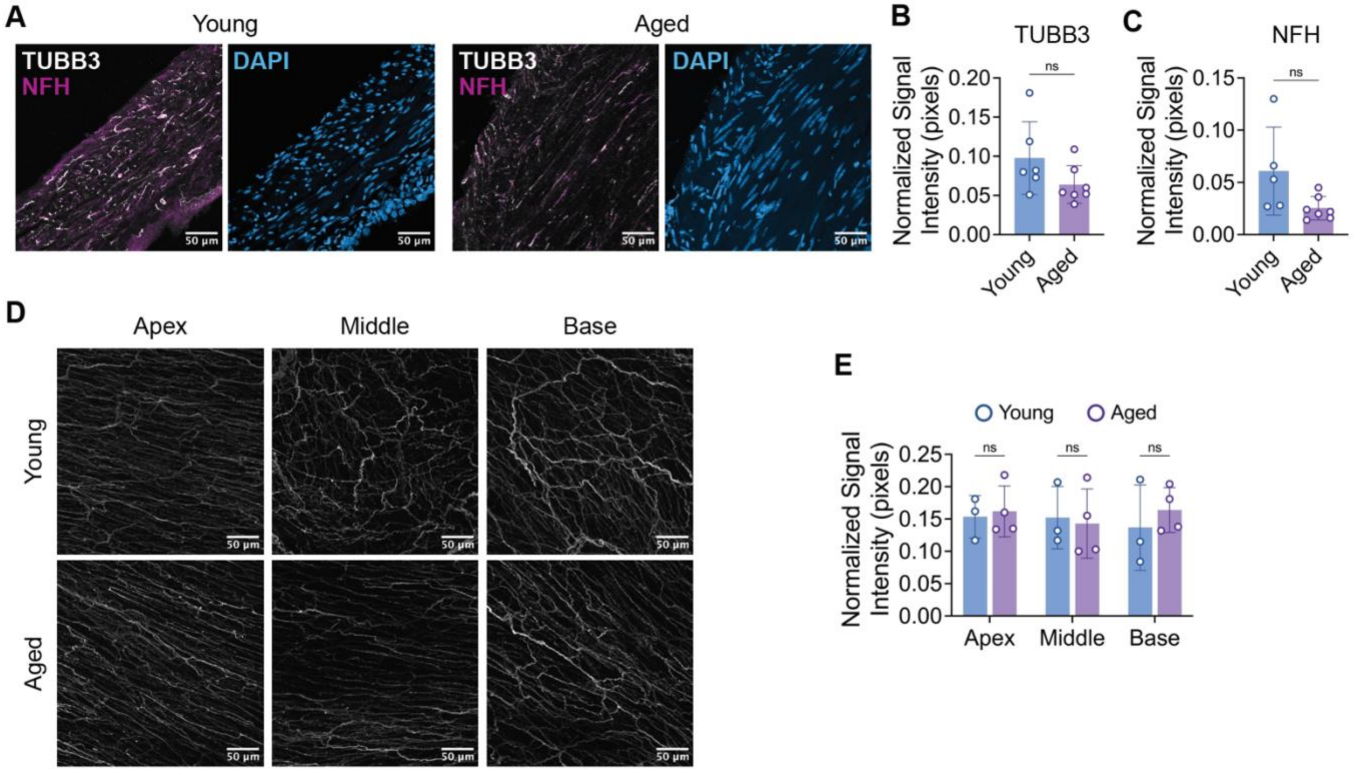

Figure S2. Bladder muscle innervation is similar in young and aged mice.

(A) Representative immunohistochemistry images from young and aged mouse bladders tissues sections, labelled against TUBB3 (pan-neuronal marker) and NFH (myelinated neurons).

(B-C) Quantification of tissue section immunohistochemistry, shown as signal pixel intensity normalized to tissue area; n=6-7/group.

(D) Representative immunostaining images from whole-mount young and aged mouse bladders labelled against TUBB3 (pan-neuronal marker).

(E) Quantification of whole-mount immunohistochemistry, shown as signal pixel intensity normalized to tissue area; n=3-4/group.

For all bar graphs: bars show mean ±SD (error bars), dots show individual animal means. Statistical tests: (B-C) Welch's *t*-test. (E) Two-way ANOVA with Šídák's multiple comparisons test. (ns) $p > 0.05$, (*) $p \leq 0.05$, (**) $p \leq 0.01$, (***) $p \leq 0.001$, (****) $p \leq 0.0001$.

bioRxiv preprint doi: https://doi.org/10.64898/2026.05.24.726025; this version posted May 25, 2026. The copyright holder for this preprint (which was not certified by peer review) is the author/funder, who has granted bioRxiv a license to display the preprint in perpetuity. It is made available under a CC-BY 4.0 International license.

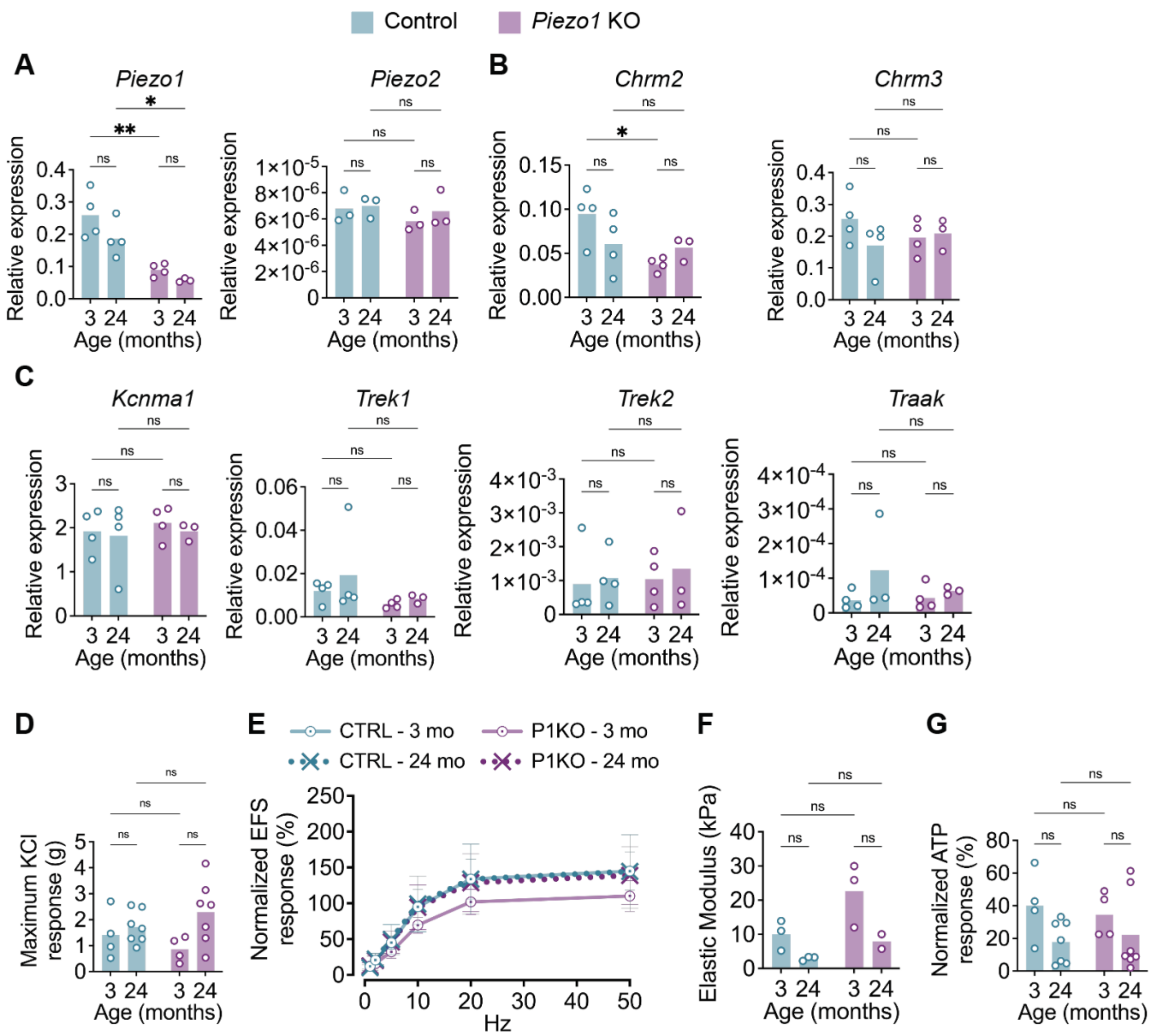

Figure S3. Smooth muscle-specific deletion of *Piezo1* alters bladder muscle properties.

(A-C) Relative mRNA expression levels of select genes in bladder smooth muscle tissues, normalized to reference genes. (A) *Piezo1* and *Piezo2* ion channels; (B) muscarinic receptors; (C) major potassium channels. n=3-4/group.

(D) KCl-induced maximal bladder muscle contractile response; n=4-7/group.

(E) Bladder muscle contractile response to electric field stimulus (EFS) at frequencies of 1, 2, 5, 10, 20, and 50 Hz; normalized to KCl-induced maximal response; n=4-7/group.

(F) Elastic modulus of isolated bladder muscle strips; n=2-3/group.

(G) Bladder muscle contractile response to 1 mM ATP, normalized to KCl-induced maximal response; n=4-7/group.

For all bar graphs: bars show mean; dots show individual animals; for (E): dots show means ±SD (error bars). Statistical tests: (A-D, F-G) Two-way ANOVA with Šídák's multiple comparisons test; (E) Two-way mixed-effects model (REML) with Tukey's multiple comparisons test; no significance bars = not significant. (ns) $p > 0.05$, (*) $p \leq 0.05$, (**) $p \leq 0.01$, (***) $p \leq 0.001$, (****) $p \leq 0.0001$.

bioRxiv preprint doi: https://doi.org/10.64898/2026.05.24.726025; this version posted May 25, 2026. The copyright holder for this preprint (which was not certified by peer review) is the author/funder, who has granted bioRxiv a license to display the preprint in perpetuity. It is made available under a CC-BY 4.0 International license.

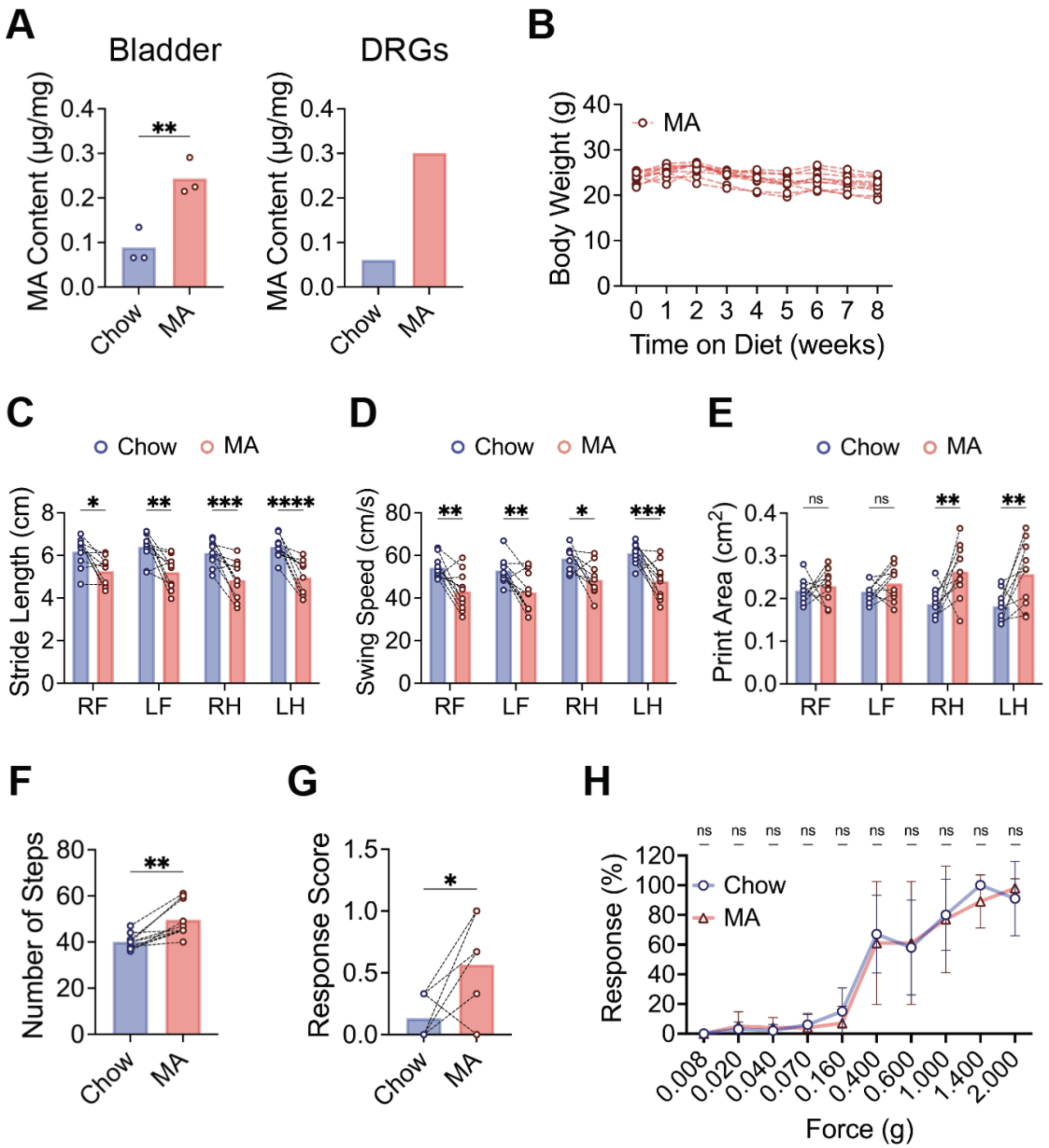

Figure S4. Characterization of systemic effects of dietary PIEZO channel manipulation in young, healthy mice

(A) Liquid chromatography-mass spectrometry analysis of margaric acid (MA) content in bladder (*left*) and DRGs tissues (*right*) from young mice fed standard chow diet (Chow, *grey*) or MA-enriched diet (MA, *red*). n=3/group; for DRG tissues, samples from 3 animals/group were pooled; bars show mean, dots represent individual animals. Statistical test for bladder tissues: Welch's *t*-test; statistical analysis for DRGs could not be performed since all 3 samples/group were pooled to meet minimum tissue weight for analysis.

(B) Chart showing body weight over 8 weeks for young mice fed MA diet. Week 0 indicates weight while on standard chow prior to the start of MA diet feeding. Individual animals shown.

(C-F) CatWalk gait analysis of young mice fed standard chow or MA diet. (C-E) Two-way repeated-measure ANOVA with Šídák's multiple comparisons test; (F-G) Paired *t*-test.

(G) Dynamic touch (brush) response scores for young mice tested at baseline (chow diet) and again after MA diet. Paired *t*-test.

(H) Von Frey touch sensitivity assay showing percentage response to each force in young mice fed chow or MA diet. Dots show mean ±SD (error bars). Two-way repeated-measure ANOVA with Šídák's multiple comparisons test.

For all bar plots: bars represent mean; dots represent individual animals. (B-I) n = 10/group. (ns) $p > 0.05$, (*) $p \leq 0.05$, (**) $p \leq 0.01$, (***) $p \leq 0.001$, (****) $p \leq 0.0001$.
